# Impact of time-of-day on immune checkpoint inhibitor therapy in cancer patients: an up-to-date clinical review

**DOI:** 10.3389/fimmu.2026.1837812

**Published:** 2026-05-29

**Authors:** Cristina Bacalam, Irina Puscariu, Daniel Sur, Claudia Burz

**Affiliations:** 1 Department of Head and Neck Oncology, Gustave Roussy, Villejuif, France; 2 Department of Medical Oncology, The Oncology Institute “Prof. Dr. Ion Chiricuţă”, Cluj-Napoca, Romania; 3 Department of Medical Oncology, Iuliu Hațieganu University of Medicine and Pharmacy, Cluj-Napoca, Romania; 4 Department of Clinical Immunology and Allergology, Iuliu Hatieganu University of Medicine and Pharmacy of Cluj, Cluj-Napoca, Romania

**Keywords:** cancer, chrono-immunotherapy, circadian rhythm, clinical review, CTLA-4, immune checkpoint inhibitors, PD-1, PD-L1

## Abstract

Over the last decade, immune checkpoint inhibitors (ICIs) have become a cornerstone of the treatment of multiple cancer types. Several factors influence ICI efficacy and toxicity, and current research is investigating whether circadian timing of administration is one of them. This review is limited in scope to ICIs (anti–CTLA-4, anti–PD-1, anti–PD-L1, anti–LAG-3) and does not detail other immunotherapeutic modalities (oncolytic viruses, cytokine therapies, adoptive cell transfer, cancer vaccines). We synthesised the findings, which are consistent with a role for the circadian rhythm in modulating normal immune function, including reported circadian variation in the activity of specific checkpoint molecules and immune cell populations. Several studies, predominantly retrospective, have reported associations between earlier-in-the-day ICI infusion and more favourable efficacy outcomes; however, the evidence base is heterogeneous, and the first randomised phase III trial currently subject to a Nature Medicine Editor’s Note suggests that time of day (ToD) of ICI administration may be associated with differences in efficacy, with more heterogeneous signals for toxicity. Given the risk of immortal-time bias, cycle-number confounding and scheduling bias in retrospective datasets, additional multicentre prospective randomised trials are required to establish causality and reproducibility, alongside research into circadian biomarkers that could help personalise timing in routine clinical care.

## 1 Introduction

The introduction of immunotherapy with the approval of the human cytotoxic T-lymphocyte antigen 4 (CTLA-4)-blocking antibody, ipilimumab, in March 2011 for the treatment of late-stage melanoma has redefined the treatment of multiple cancers, offering new and effective ways to combat this disease. While the term “immunotherapy” is used to refer to multiple approaches to treating malignant diseases by leveraging the immune system’s antitumoral activity, such as oncolytic virus therapies, cancer vaccines, cytokine therapies, and adoptive cell transfer, it is generally associated currently with its most successful and well-known class, immune checkpoint inhibitors (ICI). ICI has been proven to prevent disease progression or even achieve a partial or complete response in a wide array of cancer types. Moreover, ICI can be combined with classic chemotherapy or targeted therapies to achieve superior responses compared with monotherapy, as is the case in lung cancer, gastric cancer, hepatocellular carcinoma, renal cell carcinoma, and cervical cancer, among others (1).

During its development from a single cell to a malignant tumour, the neoplastic disease needs to acquire specific characteristics, known as the hallmarks of cancer, to survive and thrive in the hostile environment of the human body (2). One of these requisite characteristics is the evasion of immune destruction, which enables the cancer cell to “conceal” itself from the host’s immune system. To successfully achieve this goal of avoiding immune detection, the cancer cell exploits certain physiological mechanisms, mediated through negative regulators called immune checkpoints, whose original purpose is to regulate the immune response and maintain self-tolerance (3, 4). The most well-studied of these immune checkpoints are Cytotoxic T-lymphocyte-associated protein 4 (CTLA-4) and programmed cell death 1 (PD-1) and its ligand (PD-L1). The expression of these immune pathway components is often upregulated in the tumour microenvironment, thereby preventing T-cell antitumour activity. As a consequence of this pathological misuse, the cancer cells can multiply and thrive relatively unopposed by the body’s natural defence mechanisms (5).

ICI combats the effects of these immune checkpoints by inhibiting their action, freeing the immune system to attack and destroy previously concealed malignant cells. As mentioned above, the first approved ICI was ipilimumab, a CTLA-4 inhibitor that has proven effective in the treatment of metastatic melanoma (6). It was soon followed by the approval of the anti-PD-1 antibodies, nivolumab and pembrolizumab, for the same disease (7, 8). As of January 2024, eleven ICIs have been approved by the Food and Drug Administration (FDA) (9).

Despite the success of immunotherapy in the treatment of multiple cancer types, many patients fail to respond or develop resistance to the ICI and/or experience significant adverse effects, such as pneumonitis, hypothyroidism, hypophysitis, dermatitis, enteritis, and colitis (10). One of the proposed solutions for both improving the efficacy and decreasing the risk of unwanted adverse effects of ICIs is the application of the recent discoveries made in the domain of chronobiology to the timing and duration of the immunotherapy administration (11).

Several studies have shown a direct and complex link between the circadian rhythm and the activity of the immune system, with essential effects on immunological memory, immune cell regulation, and the inflammatory response (12). Furthermore, the disruption of the circadian clock has a significant role in the development of cancer through multiple mechanisms, including the regulation of the tumour immunity cycle, lymphocyte trafficking and infiltration, and the recognition and elimination of cancer cells (13, 14). ICIs are most often administered intravenously over 30–60 minutes, although lately there have been developed subcutaneous formulations for specific drugs of this class (e.g. atezolizumab, nivolumab, pembrolizumab) (15). Several retrospective studies have reported associations between morning, as opposed to afternoon, ICI infusion and more favourable efficacy outcomes in selected cancer populations; whether these associations are causal, and whether they generalise across regimens and tumour types, remains uncertain and is the subject of ongoing prospective investigation.

In this review, we examine the current evidence for time-of-day effects on immune checkpoint inhibitor (ICI) therapy, including anti–CTLA-4, anti–PD-1, anti–PD–L1, and anti–LAG-3 agents, together with the theoretical basis, potential benefits, clinical challenges, and future perspectives of chrono-immunotherapy. Other immunotherapeutic modalities (oncolytic viruses, cytokine therapies, therapeutic vaccines) are referred to only briefly for context.

## 2 Chrono-immunology

The circadian rhythm, through complex and still poorly understood mechanisms, influences most functions of the organism, including the regulation and response of the immune system. To adapt and react to the external changes resulting from the 24-hour cycle generated by our planet’s rotation, the human body employs a time-keeping system formed by a master clock and the peripheral clocks under its command. The master clock is found in the suprachiasmatic nucleus (SCN) in the hypothalamic region of the brain and is regulated by stimuli sent through the retino-hypothalamic pathway by the photosensitive cells of the retina (16, 17). The SCN signals to peripheral clocks in organs and tissues such as the liver, lungs, kidneys, and skin via mediators such as hormones and neuronal signals, thereby ensuring their rhythmicity and synchrony (18).

The core molecular clock, at both the cellular and molecular levels, is similar in the SCN and the periphery. It consists of two main transcriptional-translational feedback loops whose purpose is to modulate one another to create a cycle of gene expression that reflects and sustains the circadian rhythm. These loops rely on activators such as Circadian Locomotor Output Cycles Kaput (CLOCK) and Brain and Muscle ARNT-1 (BMAL1), which heterodimerize to induce the transcription of circadian clock genes and inhibitors such as PERIOD (PER1, 2, and 3) and CRYPTOCHROME (CRY1 and 2), which regulate the activation of CLOCK/BMAL1 heterodimers (19, 20). Overall, at least 14 core and 37 related circadian clock genes are involved in maintaining and regulating the circadian rhythm (21–23).

**Figure 1:**
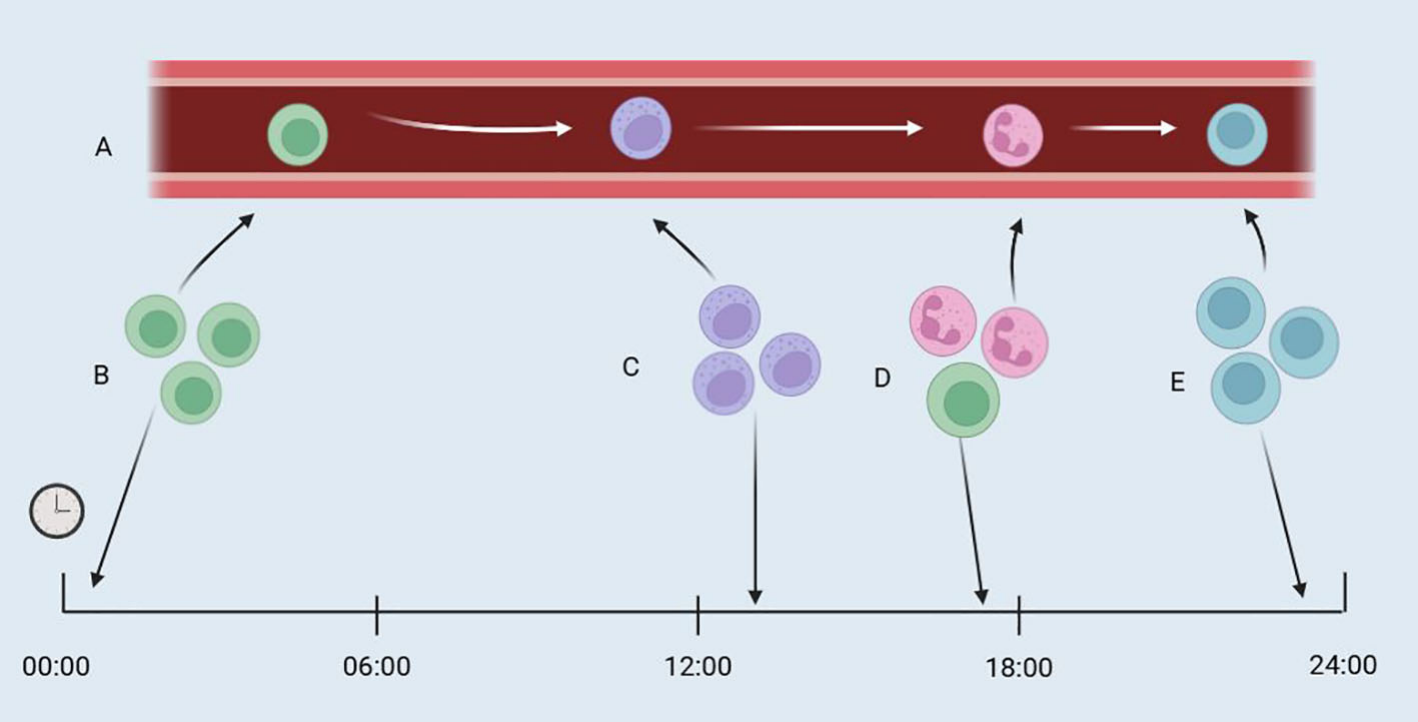
Effect of circadian rhythms on circulating immune cells. **(A)** Bloodstream. **(B)** Total (CD3+), naive (CD8+), effector, and memory (CD4+) T lymphocytes. **(C)** Natural killer cells. **(D)** Neutrophils and effector (CD8+) T lymphocytes. **(E)** B lymphocytes.

The immune system and response oscillate with the circadian rhythm through various mechanisms. Thus, pro-inflammatory cytokines (e.g. interleukin-1β (IL-1β), tumour necrosis factor-α, interferon-γ, IL-6) are considered to be somnogenic, meaning their basal plasma levels reach their peak during the rest phase (24, 25). At the opposite end of the spectrum are the anti-inflammatory cytokines, such as IL-4 and IL-10, whose levels rise following awakening and exhibit inhibitory effects on sleep (26, 27). Similarly, the composition of circulating immune cells in the bloodstream changes throughout the day, with T cells being more prominent at night and early in the morning, natural killer (NK) cells in the afternoon, neutrophils and effector T cells in the early evening, and B cells in the late evening (**Figure 1**).

Disturbances of the circadian rhythm, such as shift work and intermittent or periodic fasting, have emerged as significant factors in carcinogenesis across multiple studies (28–33). In mice subjected to conditions mimicking jet lag, an increased susceptibility to the development of lung and liver cancer has been shown (34–36). Further supporting this is the observation that many circadian clock genes and components are implicated in oncogenesis. Patients diagnosed with pancreatic ductal adenocarcinoma were found to express lower levels of PER-1, PER-2, PER-3, CRY-2, and casein kinase one isoform epsilon (CK1ϵ) (37). In hematologic malignancies, the inactivation of BMAL1 leads to disease progression by disrupting circadian clock gene expression, including C-MYC, catalase, and p300 (38). Similarly, BMAL1 plays a role in the development and metastasis of breast cancer (39, 40). All of this evidence points to the significance of circadian rhythms and their cellular mechanisms in tumorigenesis.

Another important role of the circadian rhythm is in immunological memory formation, with favourable conditions present in the early rest phase, characterised by high levels of pro-inflammatory cytokines, growth hormone and prolactin, low levels of catecholamine and cortisol, and an abundance of memory cells (41). This observation has been confirmed by preclinical and clinical data on the magnitude of the response to vaccination in the context of the time of administration (42, 43). In mice, B-cells from mice vaccinated during the rest phase produced more antibodies at 14 and 28 days than those vaccinated during the active phase (44). Similarly, humans show a weaker adaptive immune response following vaccination in the afternoon (during the active phase) compared with the morning (at the end of the rest phase) (45–48).

In the context of cancer, retrospective clinical observations have suggested that immune and tumour cells may respond differently to ICIs depending on the timing of administration, with some studies, though not all, reporting greater efficacy and, in a subset, lower toxicity with earlier-in-the-day infusions (11, 49, 50). Proposed mechanisms include circadian variation in lymph-node dendritic-cell and T-cell numbers (51) and circadian regulation of CD8+ T-cell tumour infiltration and function (52). However, the causal bridge from molecular clock biology to clinical infusion timing remains incomplete, and is likely to be tumour-, regimen- and host-dependent. Taken together, this biological and clinical evidence motivates but does not yet justify the hypothesis that aligning ICI administration with the host circadian rhythm may improve patient outcomes.

## 3 Immunotherapy drugs used in clinical practice

Immune checkpoint inhibitors (ICIs) targeting PD-1 (pembrolizumab, nivolumab), PD-L1 (atezolizumab, durvalumab, avelumab), CTLA-4 (ipilimumab, tremelimumab) and LAG-3 (relatlimab) are now used across a wide range of malignancies, including melanoma, NSCLC, renal cell carcinoma (RCC), urothelial cancer, oesophageal squamous cell carcinoma, and several hepatobiliary and gastrointestinal cancers (1, 5). Most agents are administered intravenously every 2 to 6 weeks; subcutaneous formulations are beginning to enter clinical use for selected agents (15). Treatment is usually continued until disease progression or unacceptable toxicity, frequently capped at 1–2 years in curative-intent or adjuvant settings. Importantly, the time of day (ToD) of ICI infusion was not pre-specified in any of the pivotal registration trials (53, 54). In routine clinical care, ICI infusion slots are assigned largely on logistical grounds, clinic throughput, pharmacy batching, and staff shift patterns, rather than on a chronobiological rationale (50, 55). Modalities other than ICIs (cytokines, cell-based therapy, cancer vaccines) are not discussed further in this review.

### 3.1 Time-of-day effects on checkpoint inhibitor immunotherapy

Several clinical studies have explored whether the timing of administering combinations involving immunotherapy and chemotherapy (50, 56–58), or immunotherapy and targeted agents (57, 58) is associated with differences in treatment outcomes. An emerging pattern across several retrospective studies is that earlier time-of-day infusions of ICIs are associated with more favourable patient outcomes compared with later administration. Notably, a recent meta-analysis pooled 13 studies (1,663 patients across melanoma, lung, renal, and other cancers) and reported that patients receiving ICIs predominantly in the *morning/early afternoon* experienced a lower risk of death or progression compared to those treated later in the day (pooled hazard ratio ≈0.50, *P* < 0.00001), noting substantial heterogeneity across studies (59). A narrative review of the field (11) summarised findings across real-world cohorts and concluded that early-day ICI dosing was associated with longer PFS and/or OS in several, but not all, analyses, with effect sizes varying widely. A subsequent study-level meta-analysis of 13 cohorts (1,663 patients) quantified the pooled signal more formally, reporting a pooled HR ≈ 0.50 (P < 0.00001) with substantial between-study heterogeneity and including populations that span melanoma, lung, renal and other cancers (59). These pooled observations are hypothesis-generating rather than confirmatory, and the heterogeneity is consistent with tumour-, regimen- and definition-specific effects rather than a single unified chrono-immunotherapy benefit.

Some of the earliest clinical observations relevant to chrono-immunotherapy have been reported in studies on advanced melanoma. Qian et al. (MEMOIR study) analysed 299 patients with metastatic melanoma treated with ICIs. They reported that a greater proportion of late afternoon infusions (after ~4:30 PM) was associated with poorer survival outcomes. In that cohort, receiving ≥20% of ICI doses after 4:30 PM was associated with a 1.8-fold higher risk of death (HR 1.80) and significantly shorter OS (median OS ~4.8 years for predominantly early-day treated patients *vs* not reached for mostly early-day, *P* = .023) (49). Each 20% increase in the fraction of late-day infusions was associated with approximately 30% increase in the hazard of death. Late-day treatment was also associated with lower 1-year progression-free survival (40% vs 56%) and a more significant trend toward lower complete response rates (49). Another melanoma study by Yeung et al. focused on the timing of initial infusions. In a retrospective cohort of 121 patients, those who received all of their first four ICI infusions in the afternoon (after 12:00 PM) had substantially poorer outcomes with a median OS of 5.5 months versus 24.9 months in patients who had at least one of their initial infusions in the morning (*p* < 0.001) (60). Progression-free survival was likewise shorter among patients who received afternoon initial dosing (3.3 vs 7.6 months, p=0.009) (60) exclusively. After adjusting for measured confounders, having all initial doses in the afternoon remained was associated with a 2.4-fold greater hazard of death. These findings raise the hypothesis that the timing of early immunotherapy infusions may influence melanoma outcomes, potentially by setting up a more favourable immune trajectory early during therapy (60).

Evidence from non–small cell lung cancer (NSCLC) patients often treated with anti-PD-1/PD-1/PD-L1 monotherapy or chemo-immunotherapy combinations shows patterns that are broadly consistent with observations reported in the melanoma cohorts. A bicontinental study (France and China) by Huang et al. examined inoperable NSCLC patients receiving first-line chemo-immunotherapy and stratified outcomes by infusion timing (56). In this cohort, morning immunochemotherapy (before ~11:30 AM) was associated with longer median OS compared to afternoon dosing (33.0 months vs 19.5 months) (56). This real-world study (n≈200 across two centres) provides a hypothesis-generating signal that treatment timing may be associated with survival differences in patients receiving combined chemo-ICI regimens (56). Another chronotherapeutic analysis from France (Rousseau et al., Centre Léon Bérard) retrospectively evaluated 361 metastatic cancer patients (80% with NSCLC) on ICIs (pembrolizumab, nivolumab, etc., some with chemotherapy) (50). Infusion times ranged from ~07:30 AM to 5:21 PM, and a data-derived cutoff at 11:37 AM was used to categorise “morning” vs “afternoon” dosing (50). Patients treated predominantly in the morning were observed to have a longer survival with a median OS of 30.3 months for morning infusions vs 15.9 months for afternoon (*p* = 0.0024) (50). Among patients with good performance status (PS 0–1), morning administration was associated with a median OS of 36.7 months versus 21.3 months for late-day dosing (*p* = 0.023) (50).

The observed associations with improved efficacy were accompanied by a higher incidence of low to moderate grade immune-related toxicities; grade 1–3 immune adverse events occurred in 49% of morning-treated PS0–1 patients vs 34% of afternoon-treated patients (p = 0.028) (Catozzi et al., 2023). Morning dosing was associated with higher tumour response rates (58% vs 41% objective response for PS0–1 patients, *p* = 0.027) (50), in keeping with the observed survival differences. The authors also observed that the association between later infusion timing and poorer outcomes appeared most pronounced in fitter patients (PS0–1), no clear association with benefit from morning treatment was observed among patients with poorer performance status (PS 2–3) (50), an interesting nuance raising the hypothesis that circadian modulation of immune function may be more apparent in fitter patients. Overall, these NSCLC studies provide supportive but heterogeneous evidence that earlier ICI infusions may be associated with prolonged survival and higher response rates, albeit with a potential increase in mild-to-moderate immune-related side effects.

In contrast to this, a multicentred analysis by Cortellini et al. (53) examined outcomes in 262 NSCLC patients treated with pembrolizumab at varied infusion timings and found no significant difference in survival between morning and afternoon schedules, leading the authors to caution that it was “too soon to promote morning infusions” until prospective data are available (53, 59).

Several retrospective studies in NSCLC have reported associations between earlier immunotherapy administration and more favourable outcomes, although results have not been uniform across all cohorts (11), consistent with, but not definitive proof of, the chronotherapy hypothesis (**Table 1**).

**Table 1:** Summary of the current evidence for chrono-immunotherapy, by evidence tier.

Study	Design (evidence strength)	Tumour type	N	Reported effect (with 95% CI/P where available)	Notes/cautions
Tier A. Prospective randomised evidence					
(54) (LungTIME-C01)	Randomised, open-label, single-centre phase III	Stage IIIC–IV NSCLC	210	PFS 11.3 vs 5.7 mo (HR 0.40, 95% CI 0.29–0.55; P<0.001); OS 28.0 vs 16.8 mo (HR 0.42; P<0.001).	Single-centre Chinese cohort; predominantly male; sintilimab in the majority. No significant difference in overall irAEs. Nature Medicine Editor’s Note (19 Feb 2026) flags registration-protocol inconsistencies. Independent multicentre replication required (54).
Tier B. Retrospective cohorts reporting an early-infusion benefit (hypothesis-generating)					
(49) (MEMOIR)	Retrospective single-centre	Advanced melanoma	299	Median OS not reached vs 4.8 yrs; HR 2.04 for death with late dosing.	Propensity score matched; cycle-number not primary adjustment.
(61)	Retrospective single-centre pilot	NSCLC	95	OS 34.2 vs 9.6 mo (HR 0.17); PFS 11.3 vs 3.1 mo.	Small single-centre cohort; very wide confidence intervals; likely over-estimates true effect.
(50)	Retrospective single-centre	Solid tumours (≈80% NSCLC)	361	OS 36.7 vs 21.3 mo in PS0–1 (HR 1.53 for late dosing).	Effect absent in PS 2–3; higher grade 1–2 toxicity in morning group.
(56) (bicontinental)	Retrospective multicentre	NSCLC	713	OS 33.0 vs 19.5 mo (HR 0.47).	Consistent across French and Chinese cohorts. Not to be confused with (54) (LungTIME-C01) — different authors, different design.
(62)	Retrospective single-centre	Stage IV melanoma	73	OS 38.1 vs 14.9 mo (HR 0.45).	Small cohort; baseline balance tested by χ² only.
(60)	Retrospective single-centre	Advanced melanoma	73	OS 24.9 vs 5.5 mo favouring morning.	Small cohort.
(63)	Retrospective multicentre	Metastatic RCC	135	Adjusted HR 1.69 for TTF with late dosing (P = 0.026).	Graded association with stricter late-dose thresholds.
(64)	Retrospective single-centre	Metastatic RCC	127	OS 64.8 vs 46.3 mo (HR 0.67).	
(65)	Retrospective single-centre	Oesophageal SCC	62	Superior RR, PFS and OS with morning first dose.	OS gap narrowed over full treatment course; first-3-months focus.
(66)	Retrospective multicentre	Metastatic urothelial carcinoma	92	OS 14.0 vs 6.8 mo; PFS 11.4 vs 3.6 mo.	
(67)	Retrospective multicentre	HNSCC	113	Each 20% late infusions increased OS hazard by ≈40% (HR 1.4).	Propensity-score matched.
(58)	Retrospective single-centre	Biliary tract cancer	108	OS 14.5 vs 10.1 mo (HR 1.80 for late); PFS also improved.	PSM (1:2); small cohort.
Tier C. Retrospective cohorts with null findings or signal lost after adjustment					
(53)	Retrospective multicentre	Metastatic NSCLC	262	Timing signal non-significant after adjusting for cycle number/treatment duration.	Authors: “too soon to promote morning infusions.” Critical study for cycle-number confounding.
(55)	Retrospective single-centre	Advanced NSCLC	180	Crude PFS HR 1.44; OS P = 0.09; both lost significance after cycle-number matching.	Explicit cycle-number adjustment; illustrates immortal-time and exposure-length bias.
(68)	Retrospective multicentre (Japan)	NSCLC	257	OS 16.9 vs 14.2 mo (P = 0.409); no significant difference.	Unadjusted analysis; Japanese cohort.
Janse van (69) (INSPIRE)	Retrospective single-centre	Pan-cancer	106	No significant association under any of the ToD definitions tested.	Multiple cut-offs examined; reduces concern about cherry-picked thresholds.
(70)	Retrospective cohort	Advanced melanoma	516	No effect when analysis restricted to first 4 doses; late-dosing worse only in all-dose analysis.	Direct evidence that the apparent signal can be driven by duration-of-exposure rather than ToD.
Tier D. Contradictory signal (later infusion favored)					
(71)	Retrospective multicentre	Locally advanced oesophageal SCC	183	OS significantly better when IO given AFTER 12:00 PM (HR 0.38; P = 0.013).	Direction opposite to NSCLC/melanoma literature; suggests tumour-specific circadian sensitivities.

A recent addition to the evidence base is the randomised, open-label, single-centre phase III LungTIME-C01 trial (NCT05549037), conducted by Huang Z et al. and published online in Nature Medicine on 2 February 2026 (54). It is, to our knowledge, the first prospective randomised trial to assess the impact of Time-of-Day (ToD) on immunochemotherapy outcomes in non-small cell lung cancer (NSCLC). Two hundred and ten treatment-naïve patients with stage IIIC–IV NSCLC without actionable driver mutations were randomly assigned 1:1 to receive their first four cycles of anti-PD-1 (sintilimab or pembrolizumab) plus platinum-based chemotherapy either before 15:00 (Early ToD) or after 15:00 (Late ToD). After a median follow-up of 28.7 months, the Early ToD group had a longer median PFS (11.3 months, 95% CI 9.2–13.4, vs 5.7 months, 95% CI 5.2–6.2; HR 0.40, 95% CI 0.29–0.55; P < 0.001) and a longer median OS (28.0 vs 16.8 months; HR 0.42; P < 0.001). ORR was also higher in the Early ToD group. The published report did not demonstrate a statistically significant difference in overall immune-related adverse events between arms. These results are hypothesis-confirming rather than definitive: the trial is single-centre, open-label, and predominantly enrolled male Chinese patients receiving sintilimab, and a Nature Medicine Editor’s Note (19 February 2026) has raised methodological concerns regarding inconsistencies between the registered protocol and the published manuscript. Independent, multicentre replication is therefore required before chrono-immunochemotherapy scheduling can be regarded as standard-of-care guidance.

ICI is part of the standard treatment in metastatic **renal cell carcinoma (RCC)**, and recent analyses suggest a potential association between infusion timing and outcomes. Dizman et al. (63) reported on 135 mRCC patients treated with nivolumab (± ipilimumab) at City of Hope and collaborators. Patients were grouped into “early” (<20% of infusions after 4:30 PM) vs “late” (≥20% of infusions given after 4:30 PM) cohorts (63). The objective response rate was 36.0% with predominantly early infusions vs 29.5% with late-day infusions (not statistically significant). Median time to treatment failure (TTF) appeared longer in early cohort (9.5 months vs 4.6 months for late; HR for failure = 1.40, *P* = 0.11 unadjusted) (63). After adjusting for other factors, such as age, gender, histologic subtype, etc., the association between infusion and TTF reached statistical significance after adjustment for measured covariates (HR 1.694, *P* = 0.026, favouring early infusion). The authors noted that using stricter cutoffs to define “late” infusions (i.e., even fewer late-day infusions) further increased the hazard for worse TTF and OS in the late group, raising the possibility of a graded association between the proportion of late-day infusions and outcomes (63). While the RCC data show only a modest trend in unadjusted analyses, they are consistent with the pattern that morning scheduling has been associated with longer durability of immunotherapy benefit in some analyses (63). Larger RCC studies are needed, but these findings are broadly consistent with observations reported in other immunotherapy timing studies.

Emerging data in additional tumour types show patterns that are broadly similar to those reported above. In recurrent/metastatic oesophageal squamous cell carcinoma, Nomura et al. (65) reviewed 62 patients treated with nivolumab and found that infusion timing was associated with differences in efficacy outcomes. Patients who received their first nivolumab dose before 1:00 PM were observed to have longer OS, PFS, and a higher response rate than those receiving the first dose later in the day (65). When considering most infusions in the first 3 months, early-day dosing was associated with more favourable PFS and response, though OS differences levelled out over the entire treatment course (65). These results raise the hypothesis that treatment timing may be relevant in some gastrointestinal cancers, particularly during the early phase of therapy. In contrast to this, one study of 183 patients with locally advanced oesophageal cancer reported an association with improved survival among patients receiving treatment after 12:00 PM (HR 0.38, p = 0.013) (71). In other tumour types, including metastatic urothelial cancer and gastric cancer, retrospective analyses have reported associations between earlier infusion timing and improved survival or disease control (59, 72). A small pilot in biliary tract cancer found that nivolumab given before 4:30 PM was associated with improved survival in advanced cholangiocarcinoma (58). While these smaller studies need confirmation, they contribute to a hypothesis-generating body of evidence across multiple malignancies (**Table 1**).

Taken together, the retrospective literature and the single published randomised phase III trial suggest that receiving ICI infusions in the morning or early afternoon may be associated with longer OS and PFS in selected cohorts (**Table 2**, **Table 1**), with pooled estimates in study-level meta-analysis suggesting a magnitude on the order of HR ≈ 0.5 for OS/PFS but with substantial between-cohort heterogeneity (49, 50, 54, 59–62). We emphasise that this evidence remains predominantly hypothesis-generating: most cohorts are retrospective, most use non-uniform cut-offs, and adjustment for cycle number, immortal-time bias and within-patient scheduling drift has been inconsistent (53, 55).

**Table 2:** Relevant studies in chrono-immunotherapy.

Study	Design	N	Tumour type/Regimen	ToD definition (cut-off, which infusions)	Endpoints	Confounder control (cycle-number adjustment)	Direction & effect estimate (95% CI where reported)
(54) (LungTIME-C01; NCT05549037)	Randomised, open-label, single-centre phase III	210	Stage IIIC–IV NSCLC, no driver mutations (sintilimab or pembrolizumab + platinum chemo)	Early (<3:00 PM) vs Late (≥3:00 PM) first 4 cycles	PFS (primary); OS, ORR	1:1 randomisation; stratified by PD-L1 and stage	Early-ToD benefit: PFS 11.3 vs 5.7 mo (HR 0.40, 95% CI 0.29–0.55; P<0.001); OS 28.0 vs 16.8 mo (HR 0.42; P<0.001). No significant difference in immune-related adverse events (irAEs). Nature Medicine Editor’s Note (19 Feb 2026) flags protocol-registration inconsistencies (54).
(49) (MEMOIR)	Retrospective single-centre	299	Advanced melanoma (ipilimumab, nivolumab, pembrolizumab)	Late = ≥20% of all infusions after 4:30 PM	OS	Propensity-score matching (age, PS, LDH, steroids, RT); cycle-number not primary adjustment	Shorter OS with late infusions: median OS not reached (early) vs 4.8 yrs (late); HR 2.04 for death with late dosing (49).
(61)	Retrospective single-centre pilot	95	Advanced NSCLC (nivolumab)	Morning (<12:54 PM) vs Afternoon (≥12:54 PM); all infusions	PFS, OS, ORR	Multivariable Cox; landmark analyses at 2, 4, 6 mo	Longer OS with morning dosing: OS 34.2 vs 9.6 mo (HR 0.17); PFS 11.3 vs 3.1 mo. Small single-centre cohort; wide confidence intervals (61).
(50) (CLB cohort)	Retrospective single-centre	361	Metastatic solid tumours (≈80% NSCLC; ICI ± chemo)	Morning vs afternoon at 11:37 AM cut-off; all infusions	OS, response, toxicity	Multivariable Cox; periodic mortality-risk model; cycle-number not explicitly adjusted	Longer OS in PS0–1 with morning dosing: OS 36.7 vs 21.3 mo (HR 1.53 for late). Effect not observed in PS 2–3 (50).
(56) (bicontinental)	Retrospective multicentre (French/Chinese)	713	Advanced NSCLC (ICI + chemo)	Before vs after 11:30 AM (median ToD of first 4 doses)	OS, PFS, ORR	Multivariable Cox; restricted cubic splines; consistent across cohorts	Longer OS with earlier dosing: OS 33.0 vs 19.5 mo (HR 0.47). Consistent direction in both French and Chinese cohorts (56).
(62)	Retrospective single-centre	73	Stage IV melanoma	Afternoon = ≥75% of infusions after 2:00 PM; all infusions	OS, PFS, toxicity	χ² test of independence for baseline balance; no formal cycle-number adjustment	Longer OS with morning dosing: OS 38.1 vs 14.9 mo (HR 0.45). Small cohort; limited adjustment (62).
(60)	Retrospective single-centre	73	Advanced melanoma (anti-PD-1 ± anti-CTLA-4)	Afternoon = all four initial infusions after 1:00 PM	OS, PFS	Multivariable Cox	Longer OS with morning dosing: OS 24.9 vs 5.5 mo favouring morning. Small cohort (60).
(63)	Retrospective multicentre	135	Metastatic RCC (nivolumab ± ipilimumab)	Late = ≥25% of infusions after 4:30 PM	OS, TTF, ORR	Multivariable adjustment for age, sex, histology	Earlier dosing associated with longer TTF after adjustment (HR 1.69, P = 0.026 for late); OS trend similar. Graded association with stricter late-dose thresholds (63).
(64)	Retrospective single-centre	127	Metastatic RCC (nivolumab + ipilimumab)	Late = ≥20% of infusions after 4:30 PM	OS, ORR	Kaplan-Meier; Cox regression	Longer OS with morning dosing: OS 64.8 vs 46.3 mo (HR 0.67) (Arroyave 64).
(65)	Retrospective single-centre	62	Recurrent/metastatic oesophageal SCC (nivolumab)	Morning = first infusion before 1:00 PM; first 3 months focus	OS, PFS, response rate	Comparison of baseline-matched early/late; no cycle-number adjustment	Superior RR, PFS, OS with morning first dose; OS gap narrowed over full treatment course (65).
(66)	Retrospective multicentre	92	Metastatic urothelial carcinoma (anti-PD-(L)1)	Late = ≥20% of infusions after 4:30 PM	OS, PFS, ORR	Multivariable Cox	Longer OS and PFS with morning dosing: OS 14.0 vs 6.8 mo; PFS 11.4 vs 3.6 mo (66).
(67)	Retrospective multicentre	113	HNSCC (pembrolizumab-based)	Late = ≥20% of infusions after 3:00 PM	OS, PFS	Propensity-score matching	Each additional 20% of late infusions increased OS risk (HR 1.4) (67).
(58)	Retrospective single-centre	108	Advanced biliary tract cancer (ICI ± chemo/targeted)	Late = ≥20% of infusions after 4:30 PM	OS, PFS, ORR, toxicity	PSM (1:2); multivariable Cox	Longer OS with morning dosing: OS 14.5 vs 10.1 mo (HR 1.80 for late); PFS also improved (58).
(53)	Retrospective multicentre	262	Metastatic NSCLC (pembrolizumab)	Evening = ≥20% of infusions after 4:30 PM	OS, PFS	PSM including number of cycles (explicit cycle-number adjustment)	Timing signal lost significance after adjusting for treatment duration/cycle number. Authors concluded “too soon to promote morning infusions” (53).
(55)	Retrospective single-centre	180	Advanced NSCLC (nivolumab, pembrolizumab, atezolizumab)	Late = ≥20% of infusions after 4:30 PM	OS, PFS	Multivariable including number of cycles (explicit cycle-number adjustment)	PFS significant before adjustment (HR 1.44); OS non-significant (P = 0.09); effect lost when matching for cycle number (55).
(68)	Retrospective multicentre (Japan)	257	Advanced NSCLC (nivolumab)	Morning = ≥2 of first 3 doses before 12:00 PM	OS, PFS	Unadjusted early/late comparison	No significant difference in OS (16.9 vs 14.2 mo, P = 0.409) (68).
Janse van (69) (INSPIRE)	Retrospective single-centre	106	Pan-cancer (various solid tumours)	Multiple definitions tested (≥20% after 4:30 PM; noon cut-off)	OS, PFS	Adjusted for tumour-specific cohort	No significant difference under any ToD definition examined (Janse van 69).
(70)	Retrospective cohort	516	Advanced melanoma (nivolumab, pembrolizumab, ipilimumab/nivolumab)	Late = ≥20% of all infusions after 4:00 PM; separate analyses for first 4 doses vs all doses	OS, PFS, ORR, toxicity	Multivariable; explicit separate analysis for first 4 vs all doses	No effect when using only the first 4 doses; inferior PFS/OS for late dosing only when using all-dose metric. Cautionary finding re: cycle-number/exposure-length confounding (70).
(71)	Retrospective multicentre	183	Locally advanced oesophageal SCC (IO + chemo)	Late = ≥75% of infusions after 12:00 PM	OS, pCR, radiological response	1:1 PSM; multivariable Cox	OS significantly better when IO given AFTER 12:00 (HR 0.38; P = 0.013). Direction opposite to NSCLC/melanoma literature; possibly tumour-specific (71).
(73)	Phase I trial	18	Advanced cancer (recombinant interferon-α)	Circadian-rhythm-modulated continuous venous infusion (7-day)	Toxicity, dose intensity	Continuous infusion-rate titration	Positive safety signal: better tolerance/higher achievable dose intensity vs standard schedule. Historical context only; pre-dates ICI era (73).

### 3.2 Time-of-day effects on drug toxicities

Chronotherapy broadly aims to minimize the potential adverse effects of treatments while optimizing their efficacy (74; Catozzi et al., 2023; 11). Clinical trials on chronomodulated chemotherapy have suggested a reduction in drug toxicity when administered in the morning and, in some cases, observed up to 5 times greater tolerability compared to conventional schedules (11, 50, 74). A systematic review involving 18 studies presented evidence of reduced toxicity resulting from chronomodulated chemotherapy, while efficacy was maintained or improved (75).

Similar to efficacy, the toxicity profiles of ICI might be influenced by the timing of the administration. The impact of ICI infusion timing on toxicity is more complex and shows mixed results compared to traditional chemotherapy (74). While ICIs can cause adverse events, some of which are severe (76, 77), the proposed “earlier is better for less toxicity” rule seen in some chemotherapy agents does not apply consistently.

In ICI, toxicities are generally related to a strong immune response, and it is therefore biologically plausible that several studies report higher toxicities in patients who received infusions in the morning. Catozzi et al. (2023) report that in a large cohort of patients with advanced cancers, morning ICI infusions were associated with higher toxicities (specifically, Grade 1 or 2 toxicities were more frequent in the morning group) compared to later infusions (50). This was noted to align with the observed improved efficacy in the morning group, raising the possibility that higher toxicity may correlate with a more robust immune response (50). In the study by Karaboué et al. (57) (61, Nivolumab for NSCLC), fatigue (grade 3 or 4) was less frequent in the ‘morning’ group (6.3% vs. 14.9% in the ‘afternoon’ group; p = 0.024) (61). Skin toxicities (grade 2–3) occurred more frequently in the ‘morning’ group (31.9% vs. 12.7%; p = 0.049). Skin toxicities have been shown to predict improved PFS and OS on nivolumab (61). In melanoma, when considering the first four infusions, high-grade toxicity rates were similar between early and late administration groups (70). However, when considering all infusions, receiving more infusions after 4 pm was significantly associated with lower rates of severe toxicity (70). While Grade 3–4 immune-related adverse events (irAEs) were only reported in the morning (AM) treatment group, the study found no statistically significant differences in overall, G1/G2, or G3/G4 irAEs between the AM and PM treatment groups (62). Common irAEs observed were fatigue, cutaneous, endocrine, and renal toxicities (62). Catozzi et al. found that timing-related differences in treatment tolerability were significant for patients with PS0–1 but were not consistently validated for PS2-3 (50). This might be due to a dampening or loss of proper circadian organization in patients with poorer PS. Catozzi et al. observed that ICI timing appeared to be associated with differences in treatment tolerability in women, while not being a predictor variable in men (50). Women experienced more toxicities with morning infusions, aligning with prior findings for irinotecan chronotherapy. While often used to manage irAEs and cancer-related symptoms (78), several sources highlight that the use of glucocorticoids (corticosteroids) at the initiation of ICI treatment have been associated with reduced anti-PD-L1 efficacy, potentially compromising progression-free and overall survival (74, 79). This effect has been observed in melanoma and non-small cell lung cancer patients, with higher doses (appearing to be associated with poorer outcomes) (79).

In contrast, some analyses have suggested that later administration could be linked to higher toxicity, although the evidence is limited and inconsistent. Of note, the LungTIME-C01 randomised phase III trial (54) did not demonstrate a statistically significant difference in immune-related adverse events between the Early and Late ToD arms. This inverse relationship, fewer severe immune toxicities with morning dosing, may seem counterintuitive given that morning dosing has been hypothesized to provoke a more robust immune attack on tumours. One hypothesis is that patients who respond better (with morning treatment) experience milder autoimmune side effects (like thyroiditis or rash), but avoid severe toxicities associated with disease progression or high tumour burden that can occur in less responsive (late-day treated) patients. More data are needed, but clinicians should be aware that chrono-immunotherapy could alter toxicity patterns. The slight increase in low-grade toxicity seen with morning infusions in some studies (50) might reflect more vigorous immune activation (a trade-off for better tumour control), whereas delayed dosing might risk severe events if the treatment is less effective or pharmacodynamics differ later in the day. To date, available studies have not reported a clear increase in treatment-related mortality with any timing; the safety signals mainly involve manageable immune-related effects.

This indicates a complex interplay where drugs used to manage side effects might negatively impact the primary anti-cancer treatment’s effectiveness. Many available human studies, most of which are retrospective, suggest that checkpoint inhibitor outcomes may vary according to treatment timing, with several reporting more favourable outcomes when therapy is administered earlier in the day.

## 4 Discussion

The field of chrono-immunotherapy is understudied and underexplored, but its application in clinical practice has the potential to offer clinically meaningful benefits for some patients treated with immunotherapy across selected cancer types. The purpose of this review is to summarize and discuss the most recent findings and perspectives about its theoretical basis, its implementation in clinical practice, and its potential implications for cancer treatment. The rationale for chronotherapy is informed by evidence suggesting that circadian rhythms modulate the numbers, trafficking, and function of immune cells, as well as circadian variation in the expression of immunotherapy targets such as PD-1 and PD-L1 (11). Circadian disruption has been associated with tumour growth and immune evasion in preclinical and observational studies (13, 35, 79–81).

A significant body of retrospective data, alongside the first randomised trial by Huang et al. (54) in NSCLC, suggests that the Time-of-Day (ToD) of immunotherapy administration may be associated with differences in clinical outcomes (54). Several studies have reported associations between earlier (morning or early afternoon) ICIs infusions and more favourable overall survival (OS), progression-free survival (PFS), and objective response rates (ORR) in selected cancer types, including melanoma, NSCLC, renal cell carcinoma, and urothelial cancer (Arroyave 13, 49, 50, 56, 60–67). However, findings across studies have been inconsistent, with some retrospective studies, such as Rousseau et al. (55) and Iwahashi et al. (68), showing no significant association between timing and outcomes in NSCLC (55, 68). Similarly, Janse van Rensburg et al. (69) found no association in a pan-cancer cohort (Janse van 69). Interestingly, Shujie Huang et al. (56) reported that for locally advanced oesophageal squamous cell carcinoma (ESCC), patients who received ≥75% of immunotherapy infusions *after* 12:00 PM had better OS, which contrasts with the “earlier is better” trend observed in most other studies. This raises the possibility of tumour-specific or drug-specific circadian sensitivities. Studies have used various cutoff times (e.g., 11:30 AM, 12:00 PM, 1:00 PM, 2:00 PM, 3:00 PM, 4:30 PM) to define “early” versus “late” infusions, which may contribute to variations in findings (Huang S et al., 2024). The most appropriate timing may be context dependent. Additionally, the efficacy of chrono-immunotherapy may depend on the primary localisation of the patient’s cancer, and therefore, the timing of administration may ultimately require individualisation, pending further prospective validation.

Several practical and biological questions arise when chrono-immunotherapy principles are applied to combination regimens. In the LungTIME-C01 trial, ICI and platinum-doublet chemotherapy were infused sequentially during the same ‘early’ or ‘late’ clinic visit, so the observed effect cannot be attributed to timing of the ICI alone; the chemotherapy component is itself known to have circadian-modulated toxicity and, possibly, efficacy (74, 75). In combination ICI regimens (e.g., ipilimumab plus nivolumab in melanoma and RCC), it remains unknown whether the relevant timing target is the first agent, the second agent, the combined infusion window, or the separation in hours between the two. In ICI plus tyrosine-kinase inhibitor regimens (e.g., nivolumab plus cabozantinib in RCC, or ICI plus lenvatinib in hepatocellular and biliary tract cancer), the TKI is given orally and daily, which further complicates any attempt to align the ICI infusion with a specific chronobiological window. Prospective trials aimed at answering these questions should pre-specify the timing of each component of the regimen rather than collapsing the administration into a single ‘early’ versus ‘late’ categorisation, and should report cycle-by-cycle timing compliance. Until such data are available, any practical recommendation about ‘morning’ scheduling of combination regimens should be framed as a pragmatic alignment with the available ICI timing literature, not as an evidence-based instruction for each component of the combination.

Despite a growing body of retrospective data and emerging randomised evidence, many articles emphasise the need for additional prospective randomised controlled trials to assess the reproducibility and robustness of these findings, to further investigate potential underlying circadian mechanisms, and to guide the potential development of personalised chronotherapy approaches (11, 74, 78). In fact, a new trial (NCT 07155317, The TIME Trial) is currently investigating whether treatment outcomes vary according to time-of-day on ipilimumab and nivolumab treatment in stage IV unresectable melanoma. This is particularly important given the potential for logistical issues, unplanned treatment delays, and selection biases inherent to retrospective studies (50, 59).

While chronotherapy has been associated with reduced toxicity for certain chemotherapies and radiotherapy regimes, the evidence for ICIs is more nuanced (73–75). Some studies suggest higher toxicities (e.g., overall Grade 1/2, skin toxicities) with earlier ICI infusions, which has been hypothesised to reflect a more robust antitumor immune response (11, 50, 61, 70). Conversely, some report less fatigue or no significant difference in overall toxicity based on infusion timing. Patient factors, such as performance status and sex, have been reported to be associated with variability in timing-related tolerability effects (50). The ongoing challenge is conducting more prospective randomised trials to better characterise timing strategies that balance efficacy and toxicity for different ICI regimens and patient populations.

Despite signals suggesting potential benefit in some studies, it is essential to note that most of them are retrospective and are therefore susceptible to multiple biases, including selection, confounding, and information bias (75, 82). Another issue with some of the retrospective studies is the small number of patients analysed, which may limit the robustness and generalizability of the conclusions. These issues may be partially addressed by synthesising available studies in meta-analysis or reviews; however, problems persist even with this approach.

The lack of a standardised cutoff time for the analysed groups, varying between 11:00 AM and 4:30 PM, makes it difficult to draw meaningful conclusions (Innominato et al., 2010; 53, 55). This variability in the timing of treatment may contribute to differences in outcomes reported across retrospective studies and may partly account for the null findings in some studies.

### 4.1 Future personalised chronotherapy

Individual chronotypes are highly relevant in chronotherapy, underscoring the necessity for personalised timing strategies (74). Chronotypes present a challenge that must be addressed before chronotherapy can be fully integrated into clinical practice (78). Circadian markers, including BMAL1 expression, glucocorticoid levels, and rest-activity patterns, may serve as predictive biomarkers for response to immunotherapy (79). Elevated BMAL1 expression in melanoma tumours correlates with improved survival following anti-PD-1 immunotherapy (74). A multifactorial biomarker panel that integrates circadian markers with established indicators such as microsatellite instability (MSI) and tumour mutational burden (TMB) may enhance the predictive value for therapeutic response (13, 52, 58, 79). Personalising therapy timing to align with an individual’s circadian cycle, for example, by utilising digital health platforms to monitor circadian markers, may enhance accuracy and effectiveness, potentially overcoming the resistance observed with conventional approaches. Given the variability in internal clocks among individuals, uniform treatment timing is unlikely to be effective. Accurate assessment methods, such as the Dim Light Melatonin Onset (DLMO) test, blood-based genomic assays, and wearable devices, are utilised to determine internal time (11, 74). Sex and age are also significant modifiers, as drug responses and optimal treatment schedules differ between men and women and change with age (11, 13, 74). Elderly patients frequently experience blunted or phase-shifted circadian rhythms, which may affect both therapeutic efficacy and side-effect profiles (13, 74). Ancestry and self-reported race may also be relevant: population-level differences have been reported in sleep duration and timing, in melatonin and cortisol rhythm amplitude, and in the allele frequency of several circadian-clock gene polymorphisms (PER3, CLOCK, BMAL1), all of which could in principle influence the magnitude and direction of a ToD effect. This is particularly relevant given that the LungTIME-C01 randomised trial was conducted in a predominantly East Asian population using sintilimab (an agent not approved in the United States or Europe), and that retrospective cohorts have drawn mainly on European and North American populations. Race, ethnicity and ancestry should therefore be routinely collected and reported in chrono-immunotherapy studies, and generalisation of the Asian population randomised trial findings to other ancestries should be made cautiously until replication data are available.

These considerations reinforce the importance of personalised timing strategies in chronotherapy to improve outcomes in cancer and other diseases. Although current evidence is promising, most chrono-immunotherapy studies are retrospective, emphasising the urgent need for prospective, randomised clinical trials to validate these findings and establish definitive clinical guidelines. Such trials should also examine inter-patient differences, including age, sex, and chronotype, as well as underlying mechanisms throughout the 24-hour cycle to support personalised scheduling. Artificial intelligence and machine learning algorithms have demonstrated potential in predicting circadian clock dysfunction and patient prognosis from single tissue samples (23, 79). This technology may be further developed to determine the optimal timing for immune checkpoint inhibitor (ICI) infusions for individual patients. For instance, the Time Teller (23) an artificial intelligence algorithm can model molecular clock function and timing from single-tissue biopsies or RNA sequencing data, identify molecular clock disruption in tumours, and potentially inform personalised treatment schedules.

## 5 Conclusions

In conclusion, circadian biology plausibly modulates innate and adaptive immune function, and a growing body of retrospective evidence, together with a single, single-centre randomised phase III trial currently subject to a Nature Medicine Editor’s Note, is compatible with the hypothesis that earlier-in-the-day ICI infusion may be associated with better efficacy outcomes in selected patient populations. This evidence base remains hypothesis-generating rather than practice-changing: the cohorts are heterogeneous, the cut-offs used to define “early” versus “late” infusion vary between studies, and the pooled effect is not robust to adjustment for cycle number in some of the largest NSCLC datasets. Independent, multicentre, prospective randomised trials are needed before chrono-immunotherapy scheduling can be recommended in routine practice. Important unresolved questions include whether timing effects differ between anti–CTLA-4 and anti–PD-(L)1 agents, whether effect is modified by patient chronotype, sex, age, ethnicity, performance status and comorbidity, and how chrono-immunotherapy interacts with the chronobiology of concurrent chemotherapy and radiotherapy. Until these questions are answered, the principal practical implication of the current evidence is that routine collection and reporting of infusion timing should become standard in immunotherapy trials and real-world datasets, so that the hypothesis can be tested rigorously rather than re-derived from retrospective records.
